# p53-independent DUX4 pathology in cell and animal models of facioscapulohumeral muscular dystrophy

**DOI:** 10.1242/dmm.030064

**Published:** 2017-10-01

**Authors:** Darko Bosnakovski, Micah D. Gearhart, Erik A. Toso, Olivia O. Recht, Anja Cucak, Abhinav K. Jain, Michelle C. Barton, Michael Kyba

**Affiliations:** 1 Lillehei Heart Institute, University of Minnesota, 312 Church St. SE, Minneapolis, MN 55455, USA; 2 Department of Pediatrics, University of Minnesota, 312 Church St. SE, Minneapolis, MN 55455, USA; 3 University Goce Delcev - Stip, Faculty of Medical Sciences, Krste Misirkov b.b., 2000 Stip, Republic of Macedonia; 4 Department of Genetics, Cell Biology and Development, University of Minnesota, 312 Church St. SE, Minneapolis, MN 55455, USA; 5 Department of Epigenetics and Molecular Carcinogenesis, The University of Texas MD Anderson Cancer Center, Houston, TX 77030, USA

**Keywords:** Facioscapulohumeral muscular dystrophy, Myopathy, DUX4, p53

## Abstract

Facioscapulohumeral muscular dystrophy (FSHD) is a genetically dominant myopathy caused by mutations that disrupt repression of the normally silent *DUX4* gene, which encodes a transcription factor that has been shown to interfere with myogenesis when misexpressed at very low levels in myoblasts and to cause cell death when overexpressed at high levels. A previous report using adeno-associated virus to deliver high levels of DUX4 to mouse skeletal muscle demonstrated severe pathology that was suppressed on a *p53*-knockout background, implying that DUX4 acted through the p53 pathway. Here, we investigate the p53 dependence of DUX4 using various *in vitro* and *in vivo* models. We find that inhibiting p53 has no effect on the cytoxicity of DUX4 on C2C12 myoblasts, and that expression of DUX4 does not lead to activation of the p53 pathway. DUX4 does lead to expression of the classic p53 target gene *Cdkn1a* (p21) but in a p53-independent manner. Meta-analysis of 5 publicly available data sets of DUX4 transcriptional profiles in both human and mouse cells shows no evidence of p53 activation, and further reveals that *Cdkn1a* is a mouse-specific target of DUX4. When the inducible DUX4 mouse model is crossed onto the *p53*-null background, we find no suppression of the male-specific lethality or skin phenotypes that are characteristic of the *DUX4* transgene, and find that primary myoblasts from this mouse are still killed by DUX4 expression. These data challenge the notion that the p53 pathway is central to the pathogenicity of DUX4.

## Abstract (precis)

**Summary:** DUX4 is thought to mediate cytopathology through p53. Here, DUX4 is shown to kill primary myoblasts and promote pathological phenotypes in the iDUX4[2.7] mouse model on the *p53*-null background, calling into question this notion.

## INTRODUCTION

Facioscapulohumeral muscular dystrophy (FSHD) affects over 25,000 people in the USA alone, making it one of the most prevalent genetic diseases. The genetic mutation underlying FSHD is usually a reduction in the copy number of a macrosatellite repeat on chromosome 4 referred to as D4Z4 (van Deutekom et al., 1993; Wijmenga et al., 1992). This repeat is GC-rich, highly methylated and normally subjected to repeat-induced silencing, which is disrupted in an allele-specific manner by contractions to 10 or fewer copies (van Overveld et al., 2003) or is disrupted on all D4Z4 repeats owing to mutation in the chromatin protein SMCHD1 (de Greef et al., 2009; Hartweck et al., 2013; Lemmers et al., 2012). When silencing at D4Z4 breaks down, an RNA transcript encoding the DUX4 protein (Gabriëls et al., 1999) is expressed. The presence of a poly(A) signal downstream of the D4Z4 repeats on chromosome 4 (chr4) (Dixit et al., 2007) leads to DUX4 expression and explains why disease is associated only with contractions of the D4Z4 repeats on chr4, and only on specific terminal 4q (4qter) alleles, so-called permissive alleles, which harbor the poly(A) signal (Lemmers et al., 2004, 2010). The DUX4 protein has been observed by immunostaining of cultured FSHD myoblasts, which show infrequent, possibly episodic, expression (Jones et al., 2012; Snider et al., 2010), and differentiated myotubes, which show more prevalent expression (Block et al., 2013; Rickard et al., 2015).

Mechanisms of DUX4-mediated pathology are currently being actively investigated. At high levels of expression, DUX4 causes toxicity that leads to cell death of myoblasts (Bosnakovski et al., 2008; Kowaljow et al., 2007), whereas, at low levels of expression, it impairs myogenic differentiation (Dandapat et al., 2014) and sensitizes cells to oxidative stress (Bosnakovski et al., 2008). A mouse model allowing doxycycline-regulated DUX4 expression has recapitulated these phenotypes in primary myoblasts (Dandapat et al., 2014) while also having several non-muscle related phenotypes due to low basal levels of DUX4 expression in the absence of doxycycline (Dandapat et al., 2016). Other animal model work includes a mouse carrying human D4Z4 repeats (Krom et al., 2013), which showed some evidence of sporadic DUX4 expression but no myopathy, and adeno-associated viral (AAV) vector-mediated delivery of DUX4 to zebrafish and mouse skeletal muscle, which showed profound myopathy (Wallace et al., 2011). This work by Wallace and colleagues implicated the p53 pathway in DUX4 pathology, as the *p53*-knockout mouse background suppressed AAV-DUX4 toxicity. The linkage to p53 is compelling because this pathway has the potential to push cells into apoptosis; however, precisely how DUX4 would activate p53 is not clear. The immediate targets of the DUX4 transcription factor include genes with regulatory elements containing the sequence TAATCTAATCA (Geng et al., 2012; Zhang et al., 2015), or variants thereof. ChIP-sequencing (ChIP-seq) has identified many genomic targets (Choi et al., 2016; Geng et al., 2012) and, at the majority of these, DUX4 displaces nucleosomes and recruits p300 and/or CBP through its C-terminus, thereby promoting acetylation of histone H3 and activation of transcription (Choi et al., 2016).

Here, we directly test the dependence on p53 of the pathogenic phenotypes of DUX4 expression by inhibiting p53 in cell lines while DUX4 is expressed, and by establishing the doxycycline-inducible iDUX4[2.7] mouse model on the *p53*-knockout background. These experiments reveal that p53 is not necessary for DUX4-mediated pathology, neither in myoblasts nor in other tissues.

## RESULTS

**Fig. 1.**
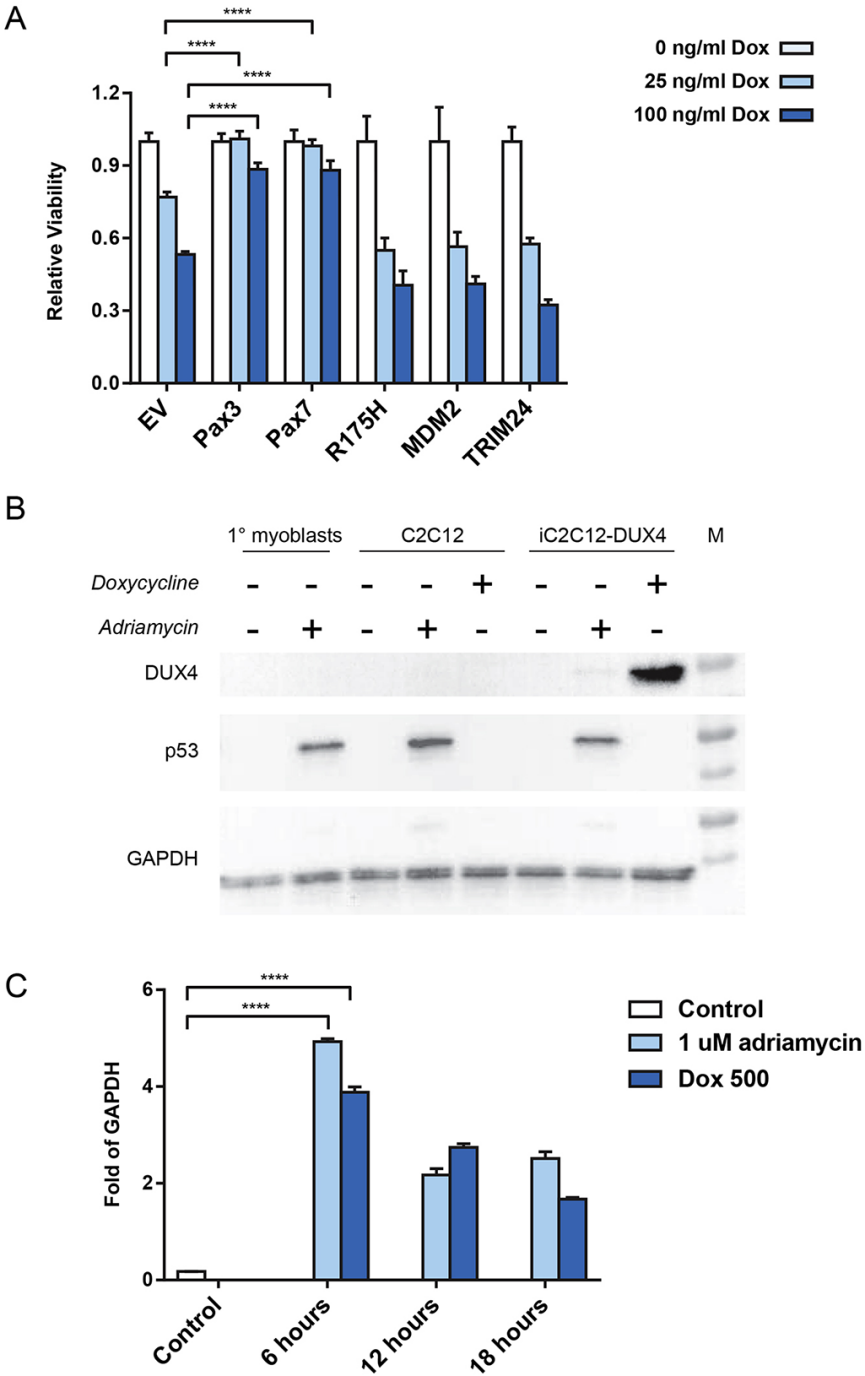
**The p53 pathway is not relevant to DUX4 activity *in vitro*.** (A) Viability of iC2C12-DUX4 cell lines, which express DUX4 in response to different doses of doxycycline and other factors constitutively from the murine stem cell virus (MSCV) long terminal repeat (LTR), or empty vector (EV). Viability in the presence of doxycycline (Dox) is normalized to that in its absence. *n*=8 biological replicates (separate wells) per group. Data presented as means±s.e.m. *t*-test: ****P<0.0001. (B) Western blots for DUX4 (top), p53 (middle) and GAPDH (bottom). Representative western blot image from 1 of 4 biological replicates is shown. (C) RTqPCR for *Cdkn1a* expression under various conditions, normalized to *Gapdh* expression. Data presented as means±s.e.m., *n*=3, *t*-test: ****P<0.0001.

Previously, overexpression of Pax3 or Pax7 was shown to reduce the toxicity of DUX4 to C2C12 mouse myoblasts (Bosnakovski et al., 2008). To see to what extent inhibiting p53 could similarly reduce DUX4 toxicity, we transduced iC2C12-DUX4 cells, which are immortalized mouse myoblasts engineered for doxycycline-inducible DUX4 expression, with retroviral vectors expressing several constructs known to interfere with p53: a dominant-negative p53 (R175H mutation), MDM2 (Momand et al., 1992) and TRIM24 (Allton et al., 2009). Overexpressing cell lines were established and cells were exposed to two different doses of doxycycline, both of which resulted in significant cell killing in the absence of overexpression (empty-vector control). Notably, although the positive controls, *Pax3* and *Pax7*, were substantially protected from apoptosis at 24 h, cells transduced with the three individual p53 inhibitors did not show a reduced death rate compared to empty vector (Fig. 1A). This indicates that, in this system, p53 is not a relevant player in the cell death phenotype.

Because C2C12 cells are an immortalized cell line, highly selected for proliferation, we investigated whether the p53 pathway was actually functional in these cells. We therefore treated C2C12 cells with Adriamycin, which causes DNA damage and induces the p53 response, and compared these to wild-type mouse myoblasts treated with Adriamycin. In both cases, Adriamycin treatment resulted in stabilization and accumulation of p53 (Fig. 1B). We also evaluated iC2C12-DUX4 cells, which were treated with Adriamycin to induce DNA damage or doxycycline to induce DUX4 expression. Although DUX4 was clearly induced by doxycycline, only Adriamycin induced a p53 response: DUX4 did not.

Finally, because the presumed linkage between DUX4 and the p53 pathway was originally suspected owing to upregulation of a strong p53 target gene, *Cdnk1a* (p21), we investigated the regulation of *Cdkn1a* in this system (Fig. 1C). We observed that both Adriamycin treatment and doxycycline treatment led to the upregulation of *Cdkn1a*. This indicates that DUX4 induces *Cdkn1a* in a p53-independent manner, i.e. *Cdkn1a* is likely a DUX4 target gene in the mouse system.

**Fig. 2.**
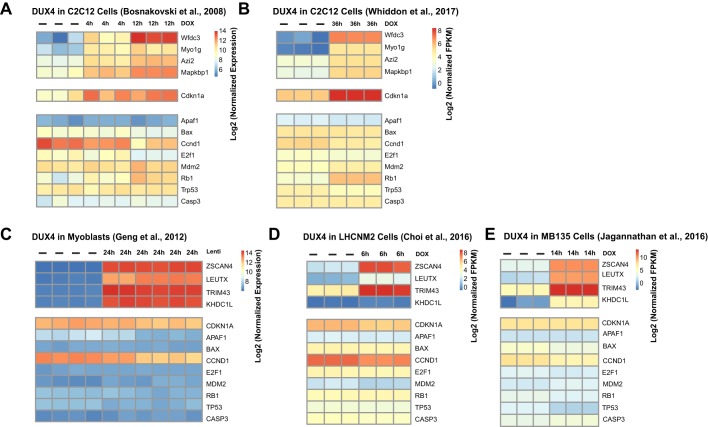
**Lack of transcriptional response to DUX4 in p53 target genes.** (A) Heatmap of log2 expression values for microarray data for DUX4 targets in mouse C2C12 cells upon doxycycline (DOX)-induced DUX4 expression. Each replicate is shown independently and the amount of time between induction and harvest is indicated above each column. DUX4 targets (upper panels) and *Cdkn1a*, which is both a DUX4 target and a p53 target in mouse cells (middle panel), are reproducibly upregulated, whereas the remaining traditional p53 targets show no discernable pattern of regulation (lower panels). (B) Heatmap of log2 expression values for RNA-seq data for DUX4 targets, grouped as in A. (C) Microarray experiments in human myoblasts cells. Gene lists are grouped as in A and B, with the exception of *Cdkn1a*, which is grouped with the p53 targets as it is not a DUX4 target in human cells. (D) RNA-seq experiments in the human LHCNM2 cell line. (E) RNA-seq experiments in the human MB135 cell line. Note that, in C, D and E, p53 targets are not induced (lower panels) despite the strong induction of DUX4 targets (upper panels). See the reference list for references stated in the figures.

To expand this analysis to a larger set of p53 pathway genes, we evaluated our and others' published transcriptional profiling data from 5 independent studies, 2 in mouse cells and 3 in human cells (Fig. 2). In each case, the set of genes annotated as DUX4 targets was found to be strongly upregulated. By contrast, the set of genes annotated as p53 targets showed no discernable upregulation, and there was even a downregulation of certain p53 targets (e.g. *Ccnd1/CCND1*, *Trp53/TP53*). This demonstrates that the published transcriptional profiling data available do not support the notion that DUX4 induces the p53 pathway.

**Fig. 3.**
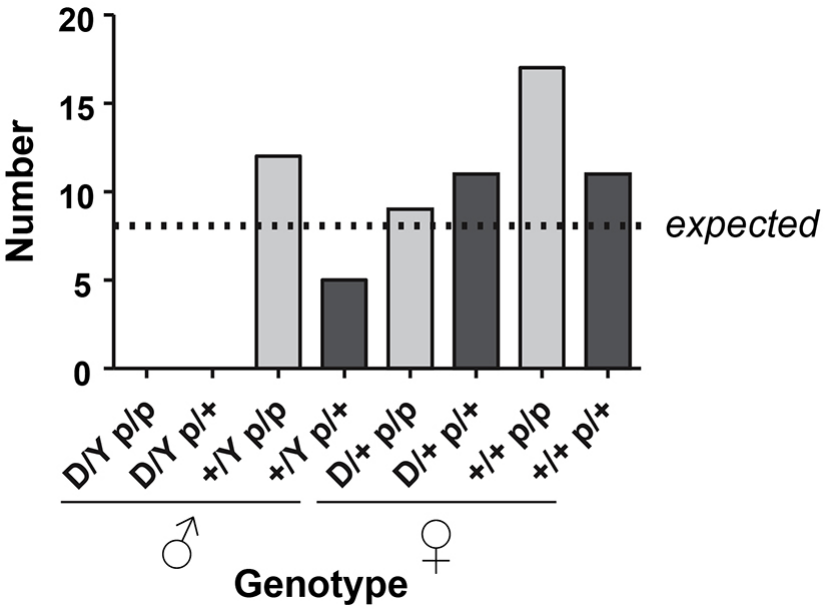
**p53 status does not impact survival of iDUX4[2.7] animals to birth.** Summary of genotypes observed from a backcross of *p53*-knockout males to *p53* heterozygous females carrying the iDUX4[2.7] transgene. Expected values are based on total numbers, assuming no loss of viability. Note that no male carriers were observed, neither in the heterozygous nor homozygous *p53*-null state. Testing the hypothesis that p53 affects survival, using the 4 classes of male progeny, *P*=0.74 (Fisher's exact test); therefore, the null hypothesis (p53 does not affect survival of iDUX4[2.7] mice) is assumed. ‘D’=presence of iDUX4[2.7] transgene, versus Y=Y-chromosome or ‘+’=wild-type X-chromosome; p=*p53*-null versus ‘+’=wild-type *p53* allele, i.e. p/p=null for *p53*.

We have previously generated a mouse model with a doxycycline-inducible 2.7-kb *DUX4* transgene (Dandapat et al., 2014, 2016), referred to as iDUX4[2.7]. One of the interesting features of this mouse is that the very low basal level of expression from the Tet-on promoter in the absence of doxycycline leads to various non-muscle phenotypes, especially in males, of which 80% die as embryos, with the remaining 20% being severely runted and all dying before 6 weeks of age. Females are less severely affected and can thus propagate the strain – because the transgene is X-linked, X-inactivation diminishes the phenotype in females. We reasoned that, if p53 were necessary for the pathological effects of DUX4 on embryonic cell types, then, on a p53 knockout background, males ought to be born at normal ratios and ought to be relatively healthy compared to siblings with a functional copy of p53. We therefore crossed the iDUX4[2.7] transgene onto the *p53*-knockout background. Female carriers were obtained that were homozygous for the *p53* knockout and these were bred to male p53 heterozygotes. We genotyped 65 progeny from this backcross and obtained no DUX4+ males, neither on the *p53*-knockout nor on the heterozygous background (Fig. 3). Thus, absence of p53 has no effect on the pathological effects of DUX4 on development in the iDUX4[2.7] strain.

Although no males were produced from the backcross described above, we did eventually obtain live-born *p53* iDUX4[2.7] knockout males from F1 crosses to Rosa-rtTA p53 double-heterozygous males, and these rare animals displayed the severe runting and skin phenotypes (flaky skin, alopecia and puffy eyelids) typical of the iDUX4[2.7] mouse (Dandapat et al., 2014). Thus, p53 is not necessary for the pathological effects of DUX4 on non-muscle tissues.

**Fig. 4.**
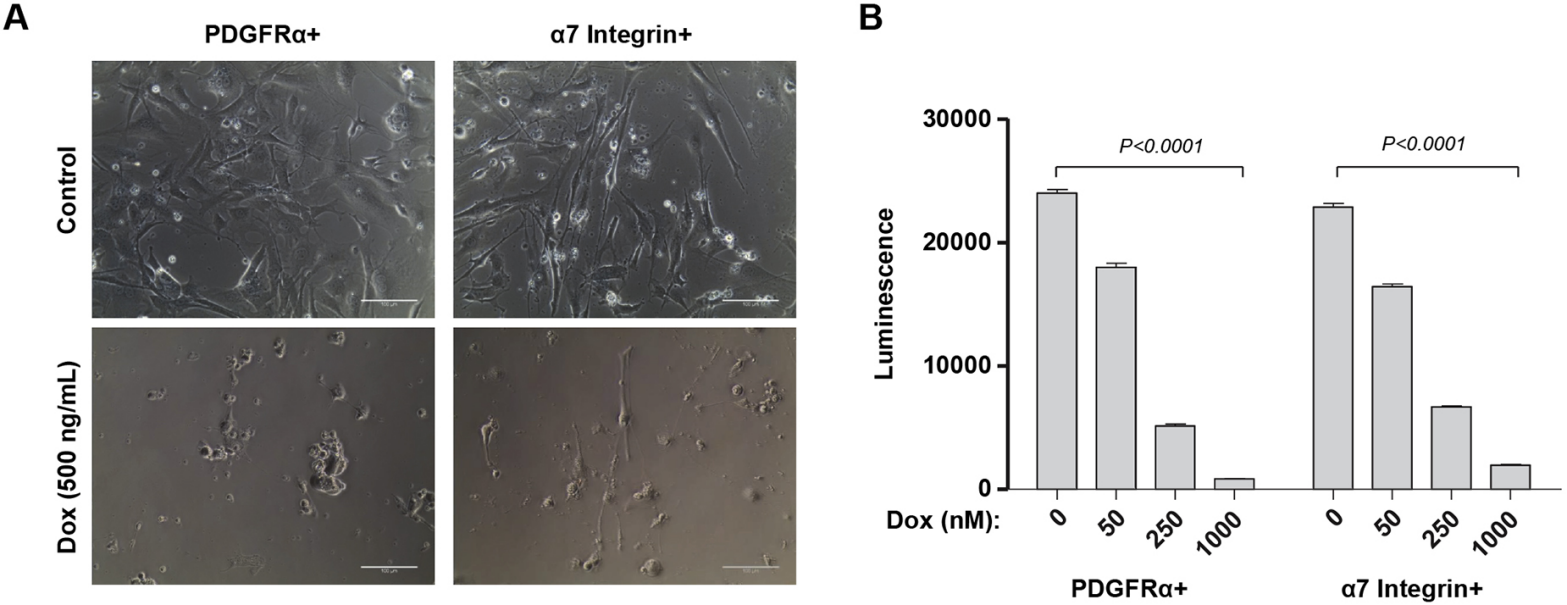
**Muscle progenitors are killed by DUX4 on the p53 mutant background.** (A) Photomicrographs of homozygous fibro/adipogenic (PDGFRα+) and myogenic (Itga7+) progenitors from male 3-week-old *p53*-knockout mice; (top) iDUX4[2.7] mice under control expansion conditions or (bottom) iDUX4[2.7] mice exposed to doxycycline (Dox) to induce DUX4 expression. Scale bars: 100 µm. (B) Viability assay for the same cells, exposed to different doses of doxycycline; *n*=8. Luminescence detects ATP, measuring viability of the cultures. Doxycycline induces loss of viability in both myoblasts and fibro/adipogenic progenitors. Data presented as means±s.e.m. (*P*<0.0001 by two way ANOVA/Sidak post hoc for both studies).

To determine whether DUX4 would be cytotoxic to muscle cells in the absence of p53, we established primary cell cultures from muscle tissue of these iDUX4[2.7]; p53 knockout animals. Primary muscle cells were sorted into myogenic and fibro/adipogenic fractions by flow cytometry for VCAM/Itga7 and PDGFRα, respectively. These sorted primary cultures were then exposed to doxycycline over a series of doses to induce DUX4 expression to different levels. DUX4 expression was clearly cytopathic (Fig. 4A) and caused a dose-dependent loss of cellular viability in myoblasts (Fig. 4B). The same was observed for fibro/adipogenic progenitors (Fig. 4A,B) on the *p53*-knockout background. Thus, in the absence of p53, DUX4 is still cytotoxic, to both myogenic and fibro/adipogenic progenitors.

## DISCUSSION

Using both the inducible C2C12 cell culture system (iC2C12-DUX4 cells), in which it was originally demonstrated that DUX4 was pathogenic to myogenic progenitors (Bosnakovski et al., 2008), and the iDUX4[2.7] mouse model (Dandapat et al., 2014), we find no evidence for p53 involvement in DUX4 pathogenicity. This lack of involvement of p53 is also supported by transcriptional profiling data from 5 independent publications. In addition, a recent report investigating shRNA suppressors of DUX4-mediated cytopathology found that a p53-knockdown vector did not suppress DUX4 pathology (Shadle et al., 2017). Interestingly, in our studies, the lack of p53 dependence applies to phenotypes caused by very low basal levels of DUX4 expression, as the developmental effects and runting are thought to be, as well as overt cytotoxicity in primary or immortalized cells, which is the result of high-level DUX4 expression.

With this in mind, it needs to be pointed out that the original study implicating p53 in DUX4 toxicity (Wallace et al., 2011) made use of a very different experimental system with vastly higher levels of DUX4 expression: recombinant AAV-vector-mediated production. The levels of DUX4 expression achieved in a cell infected with multiple copies of DUX4 driven from the CMV promoter are much higher than those in which DUX4 is expressed from its endogenous promoter from a single (terminal) D4Z4 repeat in FSHD. It is possible that, at these exceptionally high levels of expression, the p53 pathway does in fact become necessary for some aspect of extreme pathology; however, the relevance of such high levels of expression is debatable. The nature of the muscle damage seen in the AAV model is quite distinct from that seen in FSHD, and DUX4 expression is in fact quite difficult to detect in biopsies of FSHD muscle, to the point where the most reliable readout of its expression in biopsies is its target-gene fingerprint (Yao et al., 2014). It is also conceivable that some aspect of the AAV experimental system combines with the effect of DUX4 to make the pathology more severe than it would otherwise have been, and that the p53 effect ascribed to DUX4 was in fact downstream of the cellular response to AAV infection. A final difference to keep in mind is that the experiments presented in the current study do not specifically investigate p53 in adult myofibers *in vivo*. It is theoretically possible that p53 is uniquely downstream of DUX4 in adult myofibers, and thus participates in the DUX4 response only in that cell type. However, transcriptional studies on FSHD patient biopsies have not highlighted p53 as a significantly altered pathway (Celegato et al., 2006; Osborne et al., 2007; Winokur et al., 2003; Yao et al., 2014).

Regardless of the reason for the different results, the studies presented here clearly demonstrate that p53 is not necessary for the cytotoxicity of DUX4, and that the p53 pathway is not generally activated by DUX4.

## MATERIALS AND METHODS

### Mice

Animal work was conducted under a protocol approved by the University of Minnesota Institutional Animal Care and Use Committee. The *p53* mutant allele used in these studies was obtained from Jackson Laboratories (strain 008651) and carries a floxed stop codon upstream of a dominant-negative (R270H) *p53* mutant. In the absence of Cre, no functional p53 is produced; thus, as used in the current study, it is a null allele. Mice homozygous for this allele eventually succumbed to thymic lymphomas, as predicted for a *p53* null.

### Cell culture

The iC2C12-DUX4 cell line (recently validated and tested for contamination) was cultured in high-glucose Dulbecco's modified Eagle media (DMEM) supplemented with 20% fetal bovine serum (FBS, Atlanta Biologicals), L-glutamine and sodium pyruvate (Gibco), penicillin and streptomycin (from a 100× stock from Gibco) at 37°C in 5% CO_2_.

Primary mouse myoblasts and muscle fibroblasts were isolated and expanded as previously described (Arpke et al., 2013; Dandapat et al., 2014). Briefly, hindlimb muscles from 3-week-old mice were dissected under sterile conditions, minced using razor blade and digested with collagenase type II (Gibco, Grand Island, NY, 17101-015) and dispase (Gibco, 17105-041). Single-cell suspensions were plated in T75 dishes in F-10/Ham's (HyClone) medium containing 20% FBS (HyClone), 50 ng μl^−1^ human basic fibroblast growth factor (Peprotech), 1% penicillin/streptomycin (Gibco) and 1% GlutaMAX (Gibco), and cultured at 37°C at 5% O_2_, 5% CO_2_ for 5 days. From the expanded primary cells, myoblasts and fibroblasts were separated by flow cytometry for Itga7 and PDGFRα expression, respectively. Cells were trypsinized (0.25%, Gibco) and stained with PDGFRα phycoerythrin (PE) (e-Biosciences, clone: APA5, dilution: 1:200) and Itga7 allophycocyanin (APC, Ablab, R2F2, dilution: 1:200) antibodies in FACS staining medium (PBS, 1% FBS). Single-positive FACS-sorted populations were expended in the same medium and conditions used for initial primary culture.

### Expression constructs

We constructed the expression vector pMSCViGFP.GW, a Gateway-cloning-compatible retroviral expression vector, by subcloning the Gateway exchange cassette (Invitrogen) into *Xho*I/*Bgl*II-cut pMSCV-ires-GFP. The cDNAs for p53^R175H^, TRIM24 and MDM2 were amplified by PCR with flanking *attB* sites and subcloned by Gateway cloning into MSCViGFP.GW.

### Establishing overexpressing cell lines

Retroviral expression constructs were co-transfected with the packaging constructs pCL-Eco (Naviaux et al., 1996) into 293T cells using FuGENE 6 transfection reagent (Roche). Virus-containing supernatants were collected 48 h later, filtered (0.45 μm) and used directly to transduce iC2C12-DUX4 cells. Constitutively overexpressing cell lines were obtained by FACS-sorting GFP+ cells.

### Cell death inhibition assays

Cell viability was analyzed as previously described (Bosnakovski et al., 2014). Briefly, myoblasts and fibroblasts were plated in 96-well plates at 3000 cells per well in proliferation medium and treated with different concentrations of doxycycline for 72 h. Medium was removed from the plate; 100 µl working (diluted 1:3 with PBS) CellTiter-Glo luminescent assay reagent (Promega) was added to each well and luminescence, which reads out total ATP, was measured on a Cytation3 plate reader (BioTek).

### Gene expression analyses

Expression data for DUX4-expressing cells were compiled from previously published work for mouse C2C12 cells (Bosnakovski et al., 2008) and human LHCNM2 cells (Choi et al., 2016). Additional data for C2C12 (Whiddon, 2017), human myoblasts (Geng et al., 2012) and human MB135 cells (Jagannathan, 2016) were downloaded from NCBI with the accession numbers GSE87282, GSE33799 and GSE85461, respectively. RNA-seq data were mapped and quantitated as described in Choi et al. (2016). Data manipulation was performed in R (version 3.4.0) and visualizations were created with pheatmap (version 1.0.8).

### RTqPCR

RNA was isolated using Quick-RNA MiniPrep kit from Zymo Research according to the manufacturer's instructions. cDNA was synthetized from 0.75 μg of total RNA with Verso cDNA Synthesis kit (Thermo Scientific). qPCR was performed with Premix Ex Taq Master Mix (Takara) and commercially available TaqMan probes [*Gapdh*: Mm99999915_g1; *Cdkn1a* (p21): Mm00432448_m1, Applied Biosystems].

### Western blots

Western blots were performed on total proteins isolated with RIPA buffer. The following antibodies were used: rabbit anti-DUX4 (1:50 dilution, clone RD47c, R&D Systems), mouse anti-p53 (1:1000, clone 1C12, lot 4, Cell Signaling), horseradish peroxidase (HRP)-labeled mouse anti-GAPDH (1:3000, HRP-60004, Proteintech), peroxidase-conjugated goat anti-rabbit IgG (1:5000, 111-035-003, lot 127760, Jackson ImmunoResearch), goat anti-mouse IgG-HRP (1:5000, sc-2005, lot L0312, Santa Cruz Biotechnology).
